# Verbal Overshadowing of Memories for Fencing Movements Is Mediated by Expertise

**DOI:** 10.1371/journal.pone.0089276

**Published:** 2014-02-21

**Authors:** Elise Defrasne Ait-Said, François Maquestiaux, André Didierjean

**Affiliations:** 1 Université de Franche-Comté, Besançon, France; 2 UFR STAPS – Bât 335, Université Paris-Sud, Orsay, France; 3 Institut Universitaire de France and Laboratoire de Psychologie, Université de Franche-Comté, Besançon, France; VU University Amsterdam, Netherlands

## Abstract

Does verbalizing a previously-seen complex visual stimulus influence its subsequent recollection? We investigated this question by examining the mediating role played by expertise level in fencing on the effects of verbalizing upon visual memory. Participants with three distinct levels of expertise in fencing (novices, intermediates, experts) performed seven trials. In each trial, they first watched four times a short video that displayed fencing movements. Then, half of them verbalized the previously-seen visual stimulus (i.e., the verbalization group), the other half carried out a hidden-word task (i.e., the non-verbalization group). Finally, all the participants were asked to recognize the previously-seen fencing movements amongst novel fencing movements. Overall, verbalizing improved recognition for novices, altered recognition for intermediates, and had no effect for experts. These findings replicated the classical verbal-overshadowing effect, while extending it to a more conceptual material. They also point out to some potential benefits and costs of verbalizing on visual memory, depending on the level of expertise.

## Introduction

Language plays a key role in everyday life since so many of the activities that humans perform are accompanied by speech, whether directed at others or themselves. In the face of complex visual material, verbalization often helps the learner to consolidate the memory trace or build abstract knowledge [1]. However, when the material involves a type of knowledge that is highly perceptual and difficult to translate into words, such as someone’s face or the taste of a wine, verbalizing it may hinder subsequent recollection [2], [3]. However, what has to be learned is neither entirely “perceptual” nor entirely “conceptual”, but combines these two dimensions. This is the case in the art of fencing. In fencing schools, learners watch sequences of movements and are often asked to put what they see into words. This kind of learning is not only perceptual but also conceptual: learners observe and memorize a sequence of movements occurring in succession and, because each single movement composing the sequence is unique, they need to break down the sequence into its single units in order to analyze every unit in depth. The present study attempts to determine the extent to which visual memory for previously-seen sequence of fencing movements is affected by verbalization (for the influence of verbalization on motor tasks, see, [4], [5], [6], [7].

Popular beliefs, educational practices and scientific articles converge on the idea that verbalization of material to be remembered has a beneficial effect on memory and learning. It has been shown that when participants are asked to learn a novel task, verbalization during practice improves performance [8], [1], [9], [10], [11], [12], [13], [14]. These studies offered a better understanding of why verbalizing is beneficial by describing the cognitive mechanisms that enhance memorization [15] or facilitate generalization [16]. For example, Chi et al. [10] compared the benefits of various learning conditions on the acquisition of complex principles of physics. They showed that one of the most favorable conditions for learning was a situation in which two novice learners, after having watched a video of an expert solving a similar problem, had to engage in a verbal exchange about some problems they were solving.

In most of these studies, the learning of complex principles in mathematics or physics was examined. The main finding was a positive effect of verbalization. The explanation given for the verbalization effect was summarized by Gagné and Smith [17] as follow: *“It would appear that requiring verbalization somehow 'forced the subjects to think*'.” Verbalizing is thought to promote deeper processing of the material and the re-elaboration of knowledge. In this way, the material is better memorized, and abstract knowledge is built, two common objectives of learning [18].

In contrast, there are situations in which verbalizing does not always have a positive effect on learning. This is especially the case when the materiel to learn is highly perceptual. In a study on how verbalization of perceptual memories affects later recognition, Schooler and Engstler-Schooler [3] asked participants to view a short video showing a bank robbery. After a 20-minute distracting task consisting of reading a text, half of the participants had five minutes to write a detailed description of the thief’s face (verbalization group) and the other half had five minutes to carry out an hidden-word task (non-verbalization group). Finally, all the participants performed a recognition test which consisted to identify the face of the thief presented amongst seven other faces. Strikingly, the results showed that recognition accuracy was lower by 26% for the verbalization group (38% of correct recognition) than for the non-verbalization group (64% of correct recognition). Therefore, the mere act of verbalizing a difficult-to-verbalize visual stimulus altered visual memory. Schooler and Engstler-Schooler called “verbal overshadowing” this negative effect of verbalization on subsequent recognition of perceptual stimuli.

Thereafter, the verbal-overshadowing effect has been observed with other types of perceptual stimuli that are difficult to put into words: colors [3], geometric shapes [19], road maps [20], wine taste [2], voices [21], [22].

### Why Verbalizing sometimes Alters Performance

Schooler [23] proposed two hypotheses to account for the verbal-overshadowing effect in the perceptual domain: the recoding interference hypothesis and the transfer-inappropriate processing shift. According to the recoding interference hypothesis, verbalization creates a verbal code on the basis of the visual code. This verbal code is hypothesized to be imperfect due to the difficulty of using words matching the exact description of the perceptual stimulus (putting someone’s face or the taste of wine into words is often very inaccurate). At the time of recognition, verbal and visual codes would be activated and interfere with each other in working memory, thus leading to some confusion. According to the transfer-inappropriate processing shift hypothesis, verbalization triggers a shift from a global mode of processing to a more local mode of processing. For example, recognizing a face requires a global processing, whereas verbalizing causes a shift to a more analytic processing mode that focuses attention on facial details and thereby hampers later recognition.

The extent of the verbal overshadowing effect depends upon the quality of the perceptual and verbal knowledge stored in memory. More specifically, this effect is highly likely to occur in situations where the subject’s perceptual expertise is greater than her/his verbal expertise. It does not occur when the levels of perceptual and verbal expertise are comparable, whether low as is often case for novice individuals or high as is often the case for expert individuals. This is what Melcher and Schooler [2] showed in their wine-tasting experiment with wine-tasting novices, amateur wine drinkers, and wine connoisseurs. The results indicated that the act of verbalizing adversely affected recognition among those participants who had a greater perceptual expertise relative to their verbal expertise (i.e., the amateurs). However, verbalizing had no significant effect amongst the wine novices or experts. These results are consistent with the view that verbal overshadowing occurs whenever the participant’s perceptual expertise exceeds his/her verbal expertise but has no effect when the perceptual and verbal expertise levels are comparable.

### Goals and Predictions

Verbal overshadowing is an example of the negative effect of verbalization, but it only seems to occur in certain highly-specific situations [24], [25], [26]: when the stimulus is highly perceptual and when the participants’ expertise level is such that their perceptual knowledge exceeds their verbal knowledge [27], [2], [28], [29]. In the present experiment, the participants were exposed to complex visual stimuli: sequences of fencing movements that were composed of several substages such as lunging, parrying, riposte, touching. Our goal was to find out whether putting this type of complex perceptual material into words would have a beneficial or a detrimental effect on subsequent recollection, and whether the effect would depend on the learner’s expertise level. To do so, we assessed the impact of producing a verbal description of a previously-seen sequence of fencing movements on the subsequent recognition of that sequence. Participants with three levels of expertise in fencing – novices, intermediates, and experts –first watched a sequence of fencing movements, four times. Then, half of them described into words the previously-seen fencing sequence (the verbalization group), while the other half carried out an unrelated hidden-word task (the non-verbalization group). Finally, all participants had to recognize the sequence they had studied among a series of new, novel fencing sequences.

If verbal overshadowing occurs with these more-conceptual, dynamic and complex visual scenes (by analogy to what was previously shown for memory of simple perceptual stimuli such as a sip of wine or the face of a thief), we expect recognition to be poorer among intermediate-level participants who described the sequence verbally than among those who did not verbalize. No effect should be observed for experts. Novices, who have little perceptual or verbal knowledge, should perhaps benefit from verbalizing this kind of complex material (by analogy to the positive effects of verbalization found with abstract material often studied in mathematics or physics).

If, on the other hand, the negative effect of verbalizing a previous visual experience is limited solely to memory for simple perceptual stimuli, then in the face of more complex and more conceptual material, verbalizing should have no negative effect upon the memory of the participants, whatever their skill level.

## Method

### Participants

Ninety-two adults (*M* = 29.6 years, *SD* = 6.6 years, range: 19–41 years; 47 women) participated in the study. They were recruited from the local community of Besançon (Franche-Comté region) and the greater Paris area. The participants were divided into six groups on the basis of the task they would perform before the recognition test (verbalization or hidden-word task) and their fencing skill (novice, intermediate, or expert). The novices in the verbalization group (*n* = 16) and in the non-verbalization group (*n* = 15) had never fenced. The intermediates in the verbalization group (*n* = 16) and in the non-verbalization group (*n* = 15) practiced fencing as a recreational activity at a pace of two training sessions per week. The experts in the verbalization group (*n* = 15) and in the non-verbalization group (*n* = 15) were fencing instructors, top-level fencers, or modern pentathlon athletes, many of whom practiced fencing daily.

All the participants gave their written consent before participating to the experiment. The research received approval from the ethics committee of the laboratory of psychology EA 3188 (University of Franche-Comté, Besançon, France). All aspects of the research were conducted in France.

### Stimuli

Twenty-eight videos showing sequences of fencing movements were created. These sequences were presented on average for 2.75 s (*SD* = 0.79 s) and the sound was off. Each video displayed a succession of technical fencing movements performed by a master of arms and a student. The experimental procedures were controlled by programs written in E-Prime (Version 2.0) and run on Intel Pentium computers.

### Procedure

During the experiment (which lasted about 45 minutes), the participants performed seven trials. Each trial was composed of three phases. In the first, study phase, the target sequence (a video showing the fencing sequence to be learned) was repeated four times. In the second phase, one half of participants described the target sequence verbally (the verbalization group), the other half performed an unrelated, hidden-word task (the non-verbalization group). In the third, recognition phase, the target sequence and three distractive sequences (videos that were different variations of the target sequence) were presented in a random order, twice.

During the study phase, the participants were instructed to memorize the target sequence. To do so, the target sequence was presented four times. They were also instructed that they would have to recognize this sequence shortly thereafter.

During the 5-minute interval between study and recognition, half of the participants wrote a verbal description of the fencing sequence they had previously seen (verbalization group). Specifically, they were asked to write down a description of the fencing movements as accurately as possible while taking into account all elements related to body movements, weapon movements, and speed. They were also asked to write down the description in such a way that someone else reading it could unhesitatingly match the description to the video. During the same time interval, the other half of the participants carried out an unrelated hidden-word task (non-verbalization group).

During the recognition phase, the target sequence and three distractive sequences were displayed in a random order, twice. During the first presentation, the participants simply watched the four videos. During the second presentation, they had to indicate which of the four fencing sequences matched the fencing sequence they had previously studied.

## Results

**Figure 1:**
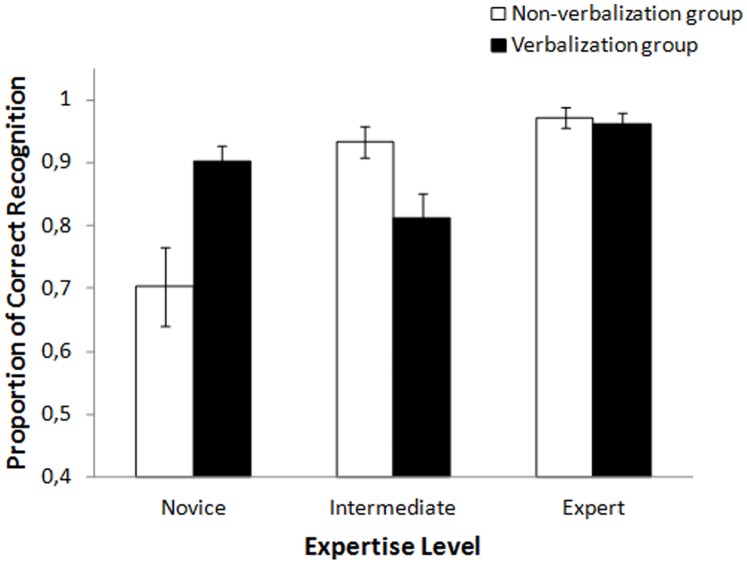
Proportion of correct recognition as a function of expertise level for the group of participants who verbalized and for the group who did not verbalize. Vertical bars show standard error.

Figure 1 shows the overall proportion of correct recognitions as a function of expertise level for the verbalization group and the non-verbalization group. An ANOVA was conducted with expertise level (novice, intermediate, or expert) and type of verbalization (non-verbalization group or verbalization group) as between-subject variables.

The results indicated a main effect of expertise level, *F*(2, 86) = 11.50, *p*<.001 (partial *η^2^* = .21). Post-hoc comparisons using the Bonferonni procedure showed that the proportion of correct recognition was larger for the experts (*M* = .97, *SD* = .06) than for the novices (*M* = .81, *SD* = .20) and the intermediates (*M* = .87, *SD* = .14), with no significant difference between the two latter groups. The main effect of type of verbalization was not significant, *F*(1, 86)<1 (partial *η^2^ = *.008).

The interaction between expertise level and type of verbalization was significant, *F*(2, 86) = 11.33, *p*<.001 (partial *η^2^* = .21). Among the novices, the proportion of correct recognition was larger for the verbalization group (*M* = .90, *SD = *.10) than for the non-verbalization group (*M* = .70, *SD* = .24), *t*(29) = 3.03, *p*<.01. Among the intermediates, the proportion of correct recognition was smaller for the verbalization group (*M* = .81, *SD* = .15) than for the non-verbalization group (*M* = .93, *SD* = .09), *t*(29) = 2.63, *p*<.05. Among the experts, the proportion of correct recognition was equivalent between the verbalization group (*M = *.96, *SD* = .07) and the non-verbalization group (*M* = .97, *SD* = .06), *t*(28)<1.

In sum, verbalizing a previously-seen sequence of fencing movements improved subsequent recollection among novices but dramatically hindered recollection among intermediates, while having no effect on recollection among experts.

As suggested by one reviewer, we also carried out a similar ANOVA as the one described in the Results section, but we added to it trial number (1, 2, 3, 4, 5, 6, or 7) as a within-subjects variable. The main effect of trial number was not significant and interacted with none of the between-subject variables, *F*s<1. Yet, if anything, the proportion of correct recognition only slightly increased from the first two trials to the last two trials, solely for the intermediates assigned to the verbalization group. This trend is in line with findings from previous studies showing that the negative effects of verbalization declined across successive trials [2], [28].

## Discussion

Our study assessed the influence of verbalization on the recognition of videos displaying sequences of fencing movements and whether this influence varied according to the level of expertise participants had in fencing. Past studies have shown that verbalization is beneficial when conceptual material has to be learned [1] but is detrimental when highly perceptual material has to be learned especially for those individuals whom the level of perceptual expertise largely exceeded the level of verbal expertise [2]. Here we used a visual material – videos displaying a sequence of fencing movements – that encompassed a large conceptual component (i.e., detecting and identifying each unit of movement composing the sequence). Our goal was to find out whether, with such a complex visual material, verbalization is positive or negative and is mediated by the participant’s level of expertise. The results indicated a contrasted effect of verbalization that depended upon expertise: verbalizing improved recognition for the novices, altered recognition for the intermediates and had no effect for the experts.

The novices had little perceptual or verbal knowledge related to fencing. In the rare studies on verbal overshadowing that have varied the participants’ expertise level, verbalization did not jeopardize performance among novices. For example, in Melcher and Schooler’s study on memory for the taste of wine [2], recognition was unaffected by verbalization for novices. Note that in this study, there was a trend that the novices who had verbalized recognized the tasted wines slightly better than those who had not. Our results with material that was more conceptual showed that verbalizing at the beginning of the acquisition process had a pronounced positive impact on later recognition of previously-seen fencing sequences.

Our results for the intermediates extend the effect of verbal overshadowing to visual material that is more conceptual than that traditionally used. At certain points in the acquisition of expertise, it is undoubtedly better not to talk about what one is learning. In contrast, the experts were not affected by verbalization. Their perceptual and verbal knowledge made recognition easy for them, regardless of whether they had verbalized.

Our findings with a preexisting expertise in fencing are consistent with those of Melcher and Schooler with a newly acquired expertise in the domain of mushroom recognition [28]. In the study by Melcher and Schooler, the participants received either some perceptual training (i.e., a task consisting in quickly categorizing a set of mushroom photographs) or some conceptual training (i.e., a lecture about mushroom structures and visible features). They found that perceptually trained recognition performance was impaired by verbalization while conceptually trained recognition performance benefited from verbalization. In line with this study, verbalization in our study likely increased conceptual expertise among novices (thus improving their subsequent recognition) while it remained insufficient to outperform the level of perceptual expertise among intermediates.

By extending the verbal-overshadowing effect to a new and more conceptual type of perceptual material (here, verbalization had a detrimental effect on visual memory for intermediate-level fencers), the present results show that at certain stages of the learning process, verbalization does not always have a positive impact. It is possible that in some domains where learning is conceptual, such as mathematics or physics, as soon as visual examples (e.g., graphs or figures) are used to present the material to be learned, verbalizing can be negative. In these domains, it may be better not to put what one is learning into words at certain intermediate skill levels. In contrast, recent findings in the motor domain showed that verbalizing a previous motor experience (golf putting) can have detrimental effects upon subsequent skilled performance [5] and, most surprisingly, upon novice performance in particular when learning conditions promoted the use of nonverbal procedural knowledge [4]; for a method manipulating the type of knowledge developed at the outset of learning, see, [30], [31]; for a review, see [32]. Future research should help gaining insight into when, for which type of task and for whom recourse to words enhances or, on the contrary, hinders learning and performance.
